# Bruising following natalizumab infusion for relapsing-remitting multiple sclerosis: a case report

**DOI:** 10.4076/1752-1947-3-8955

**Published:** 2009-08-27

**Authors:** Stylianos Gatzonis, Anna Siatouni

**Affiliations:** 1 Department of Neurosurgery, Athens Medical School, 'Evangelismos' Hospital, Ipsilantou 45-47, 10676 Athens, Greece

## Abstract

**Introduction:**

Natalizumab is a new treatment for relapsing-remitting multiple sclerosis. Because of limited experience of this treatment, medical professionals must be alert to possible side effects.

**Case presentation:**

We present a 34-year-old Caucasian woman with relapsing-remitting multiple sclerosis. She developed bruises on her legs after the first and second administrations of the new monoclonal antibody, natalizumab. Clinical and laboratory investigations revealed no hematological abnormalities. At the time of writing, she has remained on natalizumab treatment without any further side effects.

**Conclusion:**

In our patient, bruises on the lower extremities may be a benign side effect of natalizumab. This is the first documented incidence of this side effect, and the patient did not require discontinuation of natalizumab treatment.

## Introduction

Natalizumab has recently been approved as a therapy for patients with relapsing-remitting multiple sclerosis (RRMS) and with inadequate response to other therapies [1]. As new therapeutic agents have evolved in the treatment of RRMS, new risks may unfold for the patients. Here we present a female patient suffering from RRMS, who was treated with natalizumab and who developed bruises in both lower extremities following the first and second infusions of natalizumab.

## Case presentation

A 34-year-old female Caucasian patient was given natalizumab (300 mg) for RRMS (according to MacDonald's criteria) by intravenous infusion every 4 weeks; her score on the Expanding Disability Status Scale was 4.0 [2]. The patient had no other medical problems, with the exception of a mild reactive depression.

The first clinical episode in April 2001, consisted of fatigue, asthenia, a feeling of imbalance, and difficulties in fine and smooth motor coordination with the left hand. A diagnosis of multiple sclerosis was made, based on clinical and laboratory findings. The first relapse was in November 2001, at which point azathioprine was administered. She developed a rash as a result of an allergic skin reaction to azathioprine. In 2002, she had three relapses and interferon beta was administered. Because of successive relapses, she was treated with cyclophosphamide, copolymer, and mitoxantrone during the next 3 years. No remarkable disease modification was achieved. She had three to four relapses each year. During 2006, the patient had difficulty waking up in the morning but she refused to receive medical treatment because she wanted to get pregnant.

She had two relapses during the second half of 2006. During the summer of 2007, following a new severe relapse, she developed major difficulty in waking; this precipitated her decision to undertake a natalizumab treatment regimen. Since February 2007, she has been treated with escitalopram (10 mg daily) for depression.

**Figure 1:**
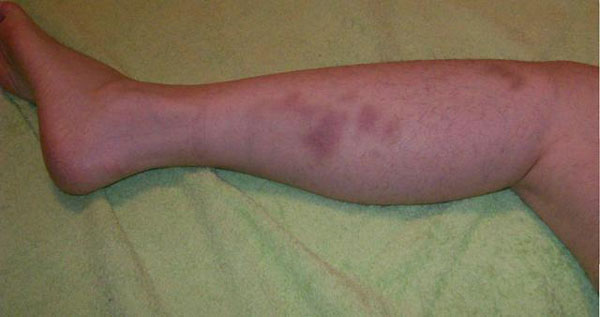
**Bruising on the external side of the left calf on the 11th day after the first natalizumab infusion**.

The patient underwent the first infusion of natalizumab on 4 September 2007. After a few hours, she presented with two small bruises on the front of her right thigh. The bruises disappeared after 3 to 4 days. However, during the 11th day, she developed bruises on the external side of her left calf (Figure 1). A detailed history of the last 10 days revealed no trauma or other mechanical reason for the bruising.

A clinical evaluation did not reveal any differences compared with the pre-infusion situation. Laboratory investigations, including blood and biochemical testing of platelet activation, liver enzymes, and thyroid hormones, as well as tests for collagen diseases and coagulation, were all normal.

There are a few reported cases that have indicated an unknown etiology of acquired FVIII inhibition, occurring in patients with MS [3]. However, in our patient, the laboratory test results for coagulation, including FVIII clotting activity, were normal.

The bruising gradually disappeared during the next 2 weeks. As a result, a detailed report was submitted to the National Medicine Organization according to Greek legislation regarding medical treatment.

One month later, she underwent the second infusion of natalizumab under close clinical and laboratory monitoring. Between the 10th and 11th day, she presented again with two small bruises on her left calf, which faded gradually and disappeared over the next 2 weeks. No other changes were noted in her clinical condition.

The third, fourth, and fifth natalizumab infusions (in November, December, and January, respectively) produced no other side effects, and no further bruising occurred.

## Discussion

According to the criteria of Karsh and Lasagna regarding cause and effect relationships in reporting adverse drug reactions, bruising on the legs in our patient could be characterized as a conditional side effect following the first and second natalizumab infusions [4]. No other clinical or laboratory findings were noteworthy.

## Conclusion

Although no clinical conclusions can be drawn from a single case, natalizumab infusion is a new treatment, and so we must pay close attention to newly occurring side effects and clinical phenomena that correlate with natalizumab administration, because their importance is still unknown.

## Abbreviation

RRMS: relapsing-remitting multiple sclerosis.

## Consent

Written informed consent was obtained from the patient for publication of this case report and any accompanying images. A copy of the written consent is available for review by the Editor-in-Chief of this journal.

## Competing interests

The authors declare that they have no competing interests.

## Authors' contributions

SG analyzed and interpreted the patient data regarding the clinical picture and treatment options. AS analyzed and interpreted the patient data regarding the hematological investigation, and was a major contributor in writing the manuscript. All authors read and approved the final manuscript.
